# 45 A Three Year Analysis of a Multi-tiered Orthotic Protocol

**DOI:** 10.1093/jbcr/irad045.019

**Published:** 2023-05-15

**Authors:** Andria Martinez, Selene Verdugo, Renee Warthman, Claudia Islas, Derek Murray, Kevin Foster

**Affiliations:** Arizona Burn Center at Valleywise Health, Peoria, Arizona; Arizona Burn Center Valleywise Health, Phoenix, Arizona; Arizona Burn Center at Valleywise Health, Phoenix, Arizona; AZ Burn Center at Valleywise Health, Phoenix, Arizona; Arizona Burn Center Valleywise Health, Phoenix, Arizona; AZ Burn Center at Valleywise Health, Cave Creek, Arizona; Arizona Burn Center at Valleywise Health, Peoria, Arizona; Arizona Burn Center Valleywise Health, Phoenix, Arizona; Arizona Burn Center at Valleywise Health, Phoenix, Arizona; AZ Burn Center at Valleywise Health, Phoenix, Arizona; Arizona Burn Center Valleywise Health, Phoenix, Arizona; AZ Burn Center at Valleywise Health, Cave Creek, Arizona; Arizona Burn Center at Valleywise Health, Peoria, Arizona; Arizona Burn Center Valleywise Health, Phoenix, Arizona; Arizona Burn Center at Valleywise Health, Phoenix, Arizona; AZ Burn Center at Valleywise Health, Phoenix, Arizona; Arizona Burn Center Valleywise Health, Phoenix, Arizona; AZ Burn Center at Valleywise Health, Cave Creek, Arizona; Arizona Burn Center at Valleywise Health, Peoria, Arizona; Arizona Burn Center Valleywise Health, Phoenix, Arizona; Arizona Burn Center at Valleywise Health, Phoenix, Arizona; AZ Burn Center at Valleywise Health, Phoenix, Arizona; Arizona Burn Center Valleywise Health, Phoenix, Arizona; AZ Burn Center at Valleywise Health, Cave Creek, Arizona; Arizona Burn Center at Valleywise Health, Peoria, Arizona; Arizona Burn Center Valleywise Health, Phoenix, Arizona; Arizona Burn Center at Valleywise Health, Phoenix, Arizona; AZ Burn Center at Valleywise Health, Phoenix, Arizona; Arizona Burn Center Valleywise Health, Phoenix, Arizona; AZ Burn Center at Valleywise Health, Cave Creek, Arizona

## Abstract

**Introduction:**

A variety of factors affect the fit of custom orthoses in those with burn injuries. A resting hand orthosis (RHO) is a common device within burn care, yet fabrication and fitting of this custom orthosis requires precision and expert assessment. However, burn therapists have varying degrees of education and credentials and previously we had noted preventable complications. Our center implemented a multi-tiered protocol focused on custom RHOs and found a marked decrease in complications immediately following implementation. The purpose of this QI project was to evaluate whether these improvements had been sustained over the following three-year period.

**Methods:**

This was a retrospective chart review. Per protocol an RHO could be fabricated by a single certified hand therapist (CHT) or co-fabricated by two (non-CHT) therapists. Incidence of orthosis complications, defined as erythema and/or an orthotic induced partial-thickness injury to the volar second metacarpal head (index finger) was the primary outcome measure. Other data collected were average time to fabricate & modify devices and average refabrication(s) required between CHT vs non-CHT devices. Patients with missing data points was excluded.

**Results:**

Eighty-five patient hands were examined from 2019-2022. Prior to implementation, total erythema occurrences were reported in 23.4% of orthoses (n=18) and total orthotic induced partial thickness injury occurred in 11.7% (n=9) of cases. Post protocol implementation erythema occurred in 5% of cases (n=5), and partial thickness injury in 0% of cases (n=0). This represents a decrease of 95% and 100%, respectively. On average CHTs fabricated RHOs in 59 minutes vs 48 minutes for non CHTs. CHTs had a complication rate of 0.0% vs 10.98% for non-CHTs. Potential complication was also limited to 0.0% for CHTS and 27% of non-CHTs. Modification rates were limited to 18.75% for CHTS vs 47.56% of non-CHTs.

**Conclusions:**

Data from this QI project showed a clinically significant decrease in erythema and decrease in breakdown and number of modifications needed from utilization of either a CHT or two therapists when managing hand orthoses. Since instituting our protocol focusing on RHOs, our center has decreased complications and potential complications to improve patient outcomes. Although, most therapists receive standard education, it can be inferred that CHTs may approach orthotic fabrication through a different lens. The required studying and education required to maintain an advanced practice certification may benefit burn therapy departments. Therapists without this specialty certification can enhance skill and outcomes via co-fabrication and assessment.

**Applicability of Research to Practice:**

Orthotic induced erythema may be decreased by utilizing CHTs or two non-CHTs to manage RHOs. This protocol can be expanded to include other orthoses or with other advanced certifications.

